# Toxicogenomics Data on Acetaminophen Now Available

**Published:** 2005-04

The National Toxicology Program (NTP), under the leadership of director Kenneth Olden and deputy director Christopher Portier, recently completed a study of hepatic transcript levels following exposure to the model hepatotoxicant acetaminophen. The study was conducted under the Principles of Good Laboratory Practice, and the pathology diagnoses were reviewed and approved following standard NTP guidelines.

The study involved 240 male F344/N rats, half of which were dosed at night. Exposure was a single oral gavage of doses varying from nontoxic to toxic, with collection of the left lobe of the liver for transcript measurements at 6, 18, 24, and 48 hours after dosing. The RNA from time-matched controls served as the reference sample, and 4 individual rats at each dose and time were hybridized against the controls using Agilent’s 60 mer rat oligo microarray chip with 20,000 features. With fluor reversals this resulted in 256 hybridizations. Hybridizations were done under a National Center for Toxicogenomics (NCT) contract. Details of the study were published in volume 33, issue 1 (2005) of *Toxicologic Pathology*.

Toxicogenomics offers the promise of the availability of large data sets that other investigators can compare against their own studies. Therefore, the NTP is making available the microarray results, individual animal pathology diagnoses, clinical chemistry, hepatic glutathione levels, and individual animal data such as body weights. These data are available at the NTP website (http://ntp.niehs.nih.gov/a/apap.html). The data will also be available in the NCT Chemical Effects in Biological Systems database, currently under development.

## Figures and Tables

**Figure:**
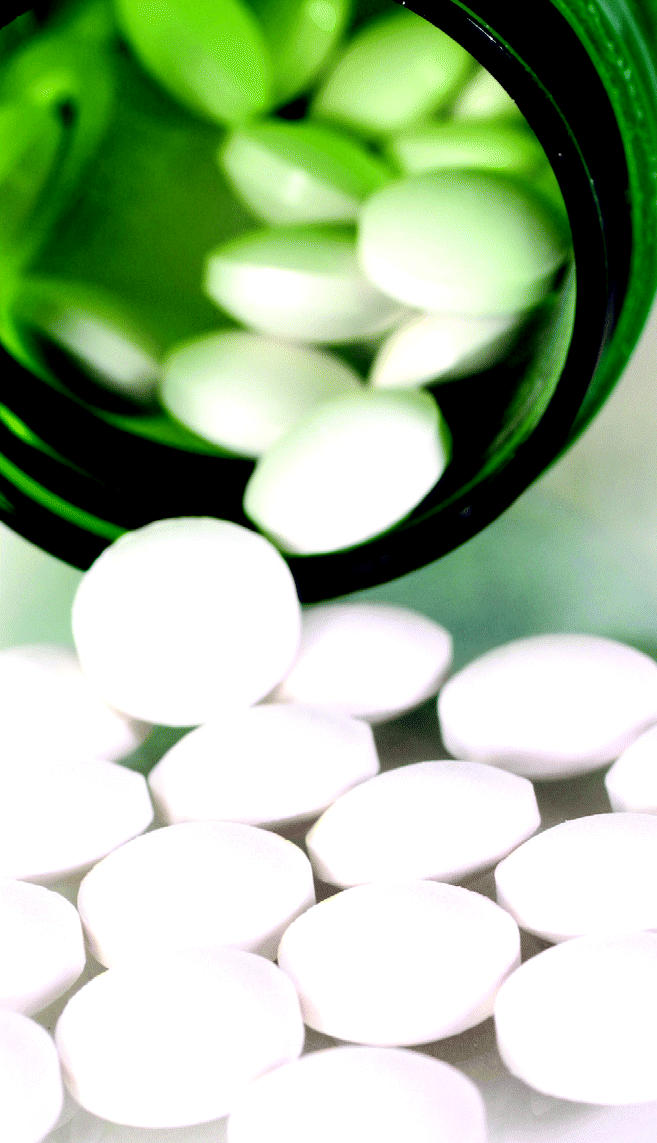
**Knowledge for all.** A broad array of toxicogenomics data on acetaminophen, a liver toxicant, is now available to any researcher.

